# Surface Electrical Stimulation for Treating Swallowing Disorders after Stroke: A Review of the Stimulation Intensity Levels and the Electrode Placements

**DOI:** 10.1155/2014/918057

**Published:** 2014-04-02

**Authors:** Marziyeh Poorjavad, Saeed Talebian Moghadam, Noureddin Nakhostin Ansari, Mostafa Daemi

**Affiliations:** ^1^Department of Speech Therapy, School of Rehabilitation, Tehran University of Medical Sciences, Enghelab Street, Tehran, Iran; ^2^Department of Physiotherapy, School of Rehabilitation, Tehran University of Medical Sciences, Tehran, Iran; ^3^Department of Speech Therapy, School of Rehabilitation, Hamedan University of Medical Sciences, Hamedan, Iran

## Abstract

Neuromuscular electrical stimulation (NMES) for treating dysphagia is a relatively new therapeutic method. There is a paucity of evidence about the use of NMES in patients with dysphagia caused by stroke. The present review aimed to introduce and discuss studies that have evaluated the efficacy of this method amongst dysphagic patients following stroke with emphasis on the intensity of stimulation (sensory or motor level) and the method of electrode placement on the neck. The majority of the reviewed studies describe some positive effects of the NMES on the neck musculature in the swallowing performance of poststroke dysphagic patients, especially when the intensity of the stimulus is adjusted at the sensory level or when the motor electrical stimulation is applied on the infrahyoid muscles during swallowing.

## 1. Introduction

Diverse paramedical treatments for swallowing disorders usually carried out by speech and language pathologists (SLPs) are introduced in the literature. It is expected that these treatment methods help to recover the swallowing functions, improve nutritional status, and prevent from developing the dysphagia consequences [1]. But, when these treatments are evaluated by scientific methods according to standards of evidence-based practice, lots of unanswered questions emerge [2] about the efficacy of them, dose-response effects, and certain populations who respond to each method well. So as mentioned by Speyer and colleagues, although some positive significant results have been published regarding the effects of different kinds of treatments in oropharyngeal dysphagia, further carefully controlled researches are needed [2].

Neuromuscular electrical stimulation (NMES) of the swallowing muscles is a relatively new therapeutic modality that is of great interest to the SLPs recently [3, 4]. Several studies were performed to evaluate the efficacy of this method. But, in spite of this increasing interest, there are important methodological issues about many of these existing publications that cause concerns regarding their therapeutic outcomes [3, 5]. A variety of protocols and techniques were used in these studies. Therefore reaching a firm conclusion about this approach and its effects will be hard. The only existing meta-analysis regarding the NMES for swallowing disorders [4] had reported just a small statistically significant improvement in clinical swallowing performance following the application of this technique. Moreover some reviews and systematic reviews [3, 5–7] emphasize the necessity for performing more carefully controlled researches. Some authors even suggest that this method should be considered premature and experimental and therefore should not be utilized in clinical settings until more relevant evidence is available [3, 5]. On the other hand, when the effects of different NMES parameters on the neural control of normal swallowing were considered, the more complicated evidences were reported. Doeltgen et al. observed a lasting increase in corticobulbar excitability at 30 and 60 minutes after electrical stimulating of the submental muscle group [8]. But these changes in corticobulbar excitability were not functionally relevant for volitional swallowing. In fact, the biomechanical swallowing studies indicated that their studied NMES protocol could lead to a swallowing that is considered less safe [9].

The present review aimed to introduce and discuss studies that have evaluated the efficacy of this method amongst dysphagic patients following stroke with emphasis on the intensity of stimulation (sensory or motor level) and the method of electrode placement on the neck. These two parameters can have important effects on the outcomes, but they had not been considered specifically by the previous published reviews [3, 5–7]. Moreover some factors and conditions toward designing further studies are proposed in this regard.

## 2. Current Intensity of Stimulation: Motor or Sensory?

The external electrical stimulation on the swallowing muscles is applied with two general purposes: to cause muscle contractions and to stimulate the sensory pathways [7]. In the first approach, the intensity of electrical current is increased until the muscle contraction occurs. These contractions may strengthen the innervated muscles [7] and also may protect the striated muscles from atrophy [10–12]. The reduced hyolaryngeal complex excursion due to weakness of the swallowing-related muscles is one of the most common abnormalities of swallowing physiology in dysphagic patients [13, 14]. Strengthening of these muscles, therefore, was recommended to improve airway protection and to increase the width of the upper esophageal sphincter (UES) opening [15, 16]. In addition, the reduced number of voluntary swallows in the tube-fed patients or patients with severe swallowing disorders can lead to disuse atrophy of swallowing-related muscles. Therefore the application of the motor surface electrical stimulation on these muscles is expected to be effective in protecting them from atrophy. However there are some differences between the pattern of motor unit recruitment in voluntary contractions and contractions induced by NMES [17]. NMES selectively activates a greater proportion of the type II muscle fibers that can produce higher levels of tension [18] and therefore will enhance strength development more [19], whereas, in voluntary contractions, the smaller type I muscle fibers are mainly activated first [17]. In some literatures [10, 11], this difference is considered as a positive point that leads to strengthening the muscles and finally to the improvement of their function. But this manner of the motor unit recruitment may prevent the outcomes from carrying over into the functional activities [17].

In the sensory approach, the sensory threshold is usually identified as the lowest current level at which the patient feels a tingling sensation on his/her neck skin [20]. Considering the sensory stimulation effects on the long-term reorganization of the human cortex [21–24], some researchers use sensory electrical stimulation to improve swallowing function.

## 3. Electrode Placement: Supra- or Infrahyoid? 

Electrode placement always is a major challenge in surface electrotherapy especially on the small and overlapping swallowing muscles. Suprahyoid muscles including the anterior belly of the digastric, the mylohyoid, and the geniohyoid pull the hyoid upward and toward the mandible. Most of the infrahyoid muscles such as sternohyoid, omohyoid, and sternothyroid muscles lower the hyolaryngeal complex toward the sternum. Therefore, when the electrodes are placed on and around the thyroid cartilage, the motor electrical stimulation will pull the larynx downward [25]. If the stimulation is applied during swallowing, this downward motion will produce a resistance against upward displacement of the hyolaryngeal structures and therefore strengthen the lifting hyolaryngeal muscles (suprahyoid muscles and thyrohyoid muscle). But, on the other hand, holding the hyolaryngeal complex down by the electrical stimulation may cause penetraion/aspiration [25]. The motor stimulation on the infrahyoid muscles at rest would be much less likely to strengthen the lifting hyolaryngeal muscles.

## 4. Review of the Literatures 

Freed et al. [10] introduced a kind of electrical stimulation technique on the neck, marketed under the name VitalStim, to elicit the suprahyoid and infrahyoid musculature contractions. They used a pair of surface electrodes positioned on the neck (bilaterally on the digastric muscles or one of them on the digastric muscle and the other on the thyrohyoid muscle ipsilaterally) to deliver the electrical current with invariant frequency at 80 Hz for a period of 60 minutes during voluntary swallows (Table 1). The intensity of stimulus was adjusted at the motor level in order to strengthen the swallowing muscles. The authors reported that this method is an effective and safe treatment for swallowing disorders induced by stroke and 98% of the patients who received electrical stimulation showed some improvement [10]. But a large number of concerns about study design, discussed by Steele et al. in detail [3, 5], undermine the validity of these results. Apart from these concerns (including concerns regarding the randomization method, the experimental bias, the validity of the used outcome measurement, and control of the spontaneous recovery) [5], there are some other considerations regarding the study of Freed and her colleagues. They used two different types of electrode placement (mentioned above) in their study (one position for each patient) without reporting the exact outcomes of each of them. The different placements will stimulate the different muscle groups and will change the swallowing physiology in different ways. Therefore it is not clear that the strengthening of which muscle groups has led to the reported improvement. Biomechanical and electrophysiological features of the swallowing process were not reported too. Moreover the authors [10] compared the motor electrical stimulation (ES) approach with the thermal-tactile stimulation (TS) approach. Since TS method is basically a sensory approach to trigger the swallowing reflex, this comparison in order to establish the efficacy of a muscle strengthening approach cannot be conclusive.

Bülow et al. [26] compared the outcomes of NMES versus a combination of traditional therapy techniques in the stroke patients with chronic dysphagia. Although patients showed significant improvement in the swallowing ability when combining both groups (*n* = 25), there were no statistically significant differences in the outcomes between groups (Table 1). Moreover the videofluorographic analysis did not show any significant therapeutic effects in the width of the UES opening, the pharyngeal delay time, the misdirection of the bolus, and the amount of the retention. The electrode placement in this study included stimulation of the thyrohyoid muscles bilaterally at the motor level. Such a configuration was shown, which lowers the hyoid and the larynx when the stimulation is applied at rest and reduces the laryngeal and hyoid peak elevation when the stimulation is used during swallowing [30]. Although Bülow et al. have not clearly said whether the stimulation was applied at rest or during swallowing, but it seems that it had been used at rest. The stimulation of the infrahyoid muscles at rest improbably leads to the strengthening of the muscles that pull the hyolaryngeal complex up. It is not unexpected that, therefore, none of the reported videofluorographic variables changed by this kind of electrical stimulation.

Permsirivanich et al. [27] compared treatment outcomes between the NMES intervention and the rehabilitation swallowing therapy (RST) in the patients with postacute (>2 wk) pharyngeal dysphagia caused by stroke. The RST protocol included a combination of diet modification, oral motor exercises, thermal stimulation, and rehabilitative swallowing techniques. Patients in the NMES group received VitalStim therapy at the motor level during voluntary swallowing along with oral motor exercises and diet modification. Two electrodes were placed midline 1 mm above and 1 mm below the thyroid notch and the third and the fourth electrodes were placed immediately superior and inferior to the first and the second ones, respectively. So they stimulated only the infrahyoid muscle group. Their results showed that both therapeutic protocols were effective in the treatment of persistent dysphagia in the stroke patients, but patients in the NMES group had a significantly greater change in their functional oral intake scale (FIOS) level (Table 1). The stimulation of the infrahyoid muscles in this study might act as a resistance against the upward motion of the hyoid during swallowing and finally led to the strengthening of the muscles that pull the hyoid up. But unfortunately the authors did not study the biomechanical events of the swallowing process or the electrophysiological characteristics of muscles. The mechanism of the reported improvement therefore remained uncertain.

Park et al. [28] studied the effectiveness of the infrahyoid motor ES and the infrahyoid sensory ES in combination with effortful swallow in poststroke dysphagic patients. In the experimental group, the intensity of stimulation led to a visible muscle contraction to depress the hyoid bone, but patients in the control group just felt a tingling sensation in their neck by a low-intensity ES (placebo stimulation). All patients, as a kind of resistance training, were instructed to perform a forceful swallow every 10 s during stimulation to elevate the hyolaryngeal complex. A significant increase in the vertical displacement of the larynx during swallowing was demonstrated in the experimental group after 12 sessions. Moreover the vertical movement of the hyoid bone and the UES opening during swallowing increased nonsignificantly in these patients. These measured biomechanical outcomes did not change in the control group. The authors concluded that the effortful swallowing coupled with the motor ES on the infrahyoid muscles can be considered as a new method to treat poststroke dysphagic patients with decreased hyolaryngeal elevation [28].

Apart from these studies that have used electrical stimulation at the motor level to strengthen the swallowing muscles, there are a few studies that have applied this modality at the sensory level. For example, Lim et al. [20] compared the effectiveness of the sensory NMES combined with the thermotactile stimulation (TTS) with the TTS alone in treating dysphagia caused by stroke. Patients in the experimental group received electrical stimulation at the sensory threshold on the supra- and infrahyoid regions and the thermotactile stimulation simultaneously. Performed assessments after 4 weeks of treatment showed a significant improvement in the swallow function scoring system [10], penetration-aspiration scale [31], and pharyngeal transit time in the experimental group in comparison to the control group. Moreover, in the experimental group, 50% of patients who had had tube feeding progressed to the oral feeding, while the tubes were removed only in 14% of patients in the control group. Finally, no adverse effects were reported by patients who received the NMES therapy (Table 1).

Gallas et al. [29] used the sensory transcutaneous electrical stimulations to test the neuromodulation hypothesis in cortical motor reorganization. They stimulated the submental muscle groups during swallowing electrically. No discomfort was reported by the stroke patients and the study results showed a significant improvement in dysphagia symptoms and swallow reaction time after only 5 sessions. Moreover aspiration and pharyngeal residue decreased significantly after stimulation. Oropharyngeal transit time, pharyngeal transit time, laryngeal closure duration, and cortical pharyngeal muscle mapping, however, did not show significant changes. The authors concluded that swallowing coordination would be improved by the sensory submental electrical stimulations during swallowing (Table 1).

## 5. Further Research

The majority of the reviewed studies [10, 20, 27–29] reported some positive treatment outcomes for the surface electrical stimulation to the neck musculature in the swallowing function of the stroke patients. If the studies are divided into two groups based on the type of stimulation (sensory and motor), the results of studies that used sensory stimulation [20, 29] seem less controversial. Both published studies [20, 29] that applied electrical stimulation at the sensory level reported some improvements in the general swallowing function. The general reduction of the swallowing problems seems less dependent on the type of the electrode placement when the stimulation is adjusted at the sensory level. The general enhancement of sensory inputs to the swallowing cortex may be enough to cause some initial reduction of the swallowing problems. If this hypothesis is true, then at least a part of positive outcomes of motor electrical stimulation in the reported studies is due to enhancement of the sensory inputs. But more studies are needed to establish the role of sensory inputs when the applied stimulation is adjusted at the motor level. Park et al. [28] compared the sensory and the motor electrical stimulation approaches. But they did not study their patients' general swallowing function or the nutritional level. Moreover some physiological variables that are expected to be influenced by the sensory stimulation, such as swallowing reaction time, were not assessed in this study [28].

The stimulation of the thyrohyoid muscles is targeted in almost all of the reviewed studies that used a motor stimulation approach [10, 26–28] in order to enhance the laryngeal elevation [6]. Of particular concern is the fact that the electrical current reaches the sternohyoid and omohyoid muscles first when the electrodes are placed on the infrahyoid muscles group, because the sternohyoid muscle is larger and closer to the surface than the thyrohyoid muscle. Since the sternohyoid and omohyoid muscles pull the hyoid bone down and back, this kind of electrode placement leads to the downward motion of the hyoid [6]. Such a movement during swallowing, as mentioned above, may produce a resistance against upward displacement of the hyolaryngeal structures and so may strengthen lifting hyolaryngeal muscles (suprahyoid muscles and thyrohyoid muscle). But, on the other hand, such an approach may be dangerous for the patients with aspiration [25]. Moreover some severe dysphagic patients cannot ever raise their larynx during minimal motoric stimulation [28]. The electrode placement on the suprahyoid muscles, therefore, may be a safer and more conservative placement method in order to achieve hyolaryngeal excursion in dysphagic patients with weak muscles and reduced hyolaryngeal elevation. But, since the active and sufficient contraction of the thyrohyoid muscle is important for airway protection during swallowing [25], strengthening of this essential muscle can be targeted by other therapeutic methods like effortful swallow and/or Shaker exercise [15, 16, 32].

The selection of participants was not based on the underlying physiological features of patients' dysphagia or aspiration in these studies. Knowing the underlying pathophysiology of the swallowing disorders is necessary to design the appropriate and specific therapeutic protocols. We cannot expect that all stroke patients with aspiration due to either reduced hyolaryngeal elevation or delayed initiation of the pharyngeal swallowing or premature loss of the bolus take advantage of the same treatment protocol. Specifically, in regard to stroke, there are some confounding factors such as site of the lesion that can change or diversify the clinical profile of swallowing disorders in different patients. An accurate and specific prescription for each dysphagic patient therefore is essential [28]. Identification and consideration of the exact underlying pathophysiology of swallowing disorders instead of the gross and global categorization (such as stroke and Parkinson's disease) will be necessary in the future studies.

As a surprising result, a large number (20–40%) of patients who suffered from other musculature disorders respond appropriately to the inactive electrotherapy devices [33, 34]. The placebo stimulators, which are identical in appearance to the real stimulators but have no electrical output to the electrodes, have been shown to decrease symptoms such as pain [34]. Therefore more attention should be given to the “placebo effect” in designing of the future studies. Finally, despite the reported great difficulties for identifying patients who can participate in these such trials [26], further randomized controlled trials in the larger groups of the stroke patients with specified and distinct pathophysiologies of dysphagia are needed.

## 6. Conclusion

The majority of the reviewed studies describe some positive effects of the NMES on the neck musculature in the swallowing performance of poststroke dysphagic patients, especially when the intensity of the stimulus is adjusted at the sensory level or when the motor electrical stimulation is applied on the infrahyoid muscles during swallowing. However, we still need to know more about the physiologic and neurologic effects of these therapeutic methods on the stroke patients' swallowing function. The identification of stimulation effects on the underlying pathophysiology of the swallowing disorders and on the central nervous system organization will help to design specific and individual treatment protocols.

## Figures and Tables

**Table 1:** A summary of reviewed studies.

Authors	Study design	Number of subjects in groups	Site of lesion	Number of sessions in groups	Reported stimulation parameters	Reported electrode location	Outcomes
Freed et al. (2001) [10]	Controlled study	Group 1 (ES^a^): *n* = 63 stroke patients Group 2 (TTS^b^): *n* = 36 stroke patients	*Group 1 Brainstem = 7 Hemispheric = 29 Multiple strokes = 24 *Group 2 Brainstem = 1 Hemispheric = 19 Multiple strokes = 8	Group 1: 5.5 sessions (mean) Group 2: 6.0 sessions (mean) (*P* = 0.36)	Frequency: 80 Hz Pulse width: 300 *μ*s Intensity: 2.5 to 25 mA Duration: 60 minutes a day for 5 days a week Motor level	Digastric and thyrohyoid muscles	Group 1. Initial swallow score in SFSS^c^ = 0.76 ± 1.04 Posttreatment swallow score in SFSS = 4.52 ± 1.69 (*P* = 0.0048) Group 2. Initial swallow score in SFSS = 0.75 ± 1.20 Posttreatment swallow score in SFSS = 1.39 ± 1.13 (*P* < 0.0001)

Bülow et al. (2008) [26]	Randomized trial	Group 1 (ES): *n* = 12 stroke patients (>3 months) Group 2 (TDT): *n* = 13 stroke patients (>3 months)	Group 1: only hemispheric stroke Group 2: only hemispheric stroke	Group 1: 15 sessions Group 2: 15 sessions	Intensity: 4.5 to 25 mA Duration: 60 minutes a day for 5 days a week over 3 weeks Motor level	Thyrohyoid muscles	Group 1. Initial median score in ANS^g^ = 2.5 Posttreatment median score in ANS = 1.5 Group 2. Initial median score in ANS = 3 Posttreatment median score in ANS = 3

Permsirivanich et al. (2009) [27]	Randomized controlled study	Group 1 (ES + oral motor exercises): *n* = 12 patients with postacute stroke (>2 wk) Group 2 (RST^h^): *n* = 11 patients with postacute stroke (>2 wk)	NR	Group 1: 17.25 ± 5.64 sessions (mean ± SD) Group 2: 18.36 ± 3.23 sessions (mean ± SD) (*P* = 0.57)	Frequency: 80 Hz Pulse duration: 700 ms Duration: 60 minutes a day for a 5 days a week Motor level	Thyrohyoid muscles	Group 1. Average changes in FOIS^i^ score = 3.17 ± 1.27 Group 2. Average changes in FOIS score= 2.46 ± 1.04 (*P* < 0.001)

Park et al. (2012) [28]	Randomized controlled study	Group 1 (effortful swallow + motor ES): *n* = 9 patients with stroke (>1 month) Group 2 (effortful swallow + sensory ES): *n* = 9 patients with stroke (>1 month)	NR	12 sessions	Group 1: Frequency: 80 Hz Pulse width: 700 *μ*s Intensity: 7.33 ± 1.12 mA Duration: 20 min per week for 4 weeks Motor level Group 2: Frequency: 80 Hz Pulse width: 700 *μ*s Intensity: 2.89 ± 1.05 mA Duration: 20 min per week for 4 weeks Sensory level	Sternohyoid muscles	Group 1. A significant increase in the vertical movement of the larynx (*P* < 0.05) A nonsignificant increase in the vertical movement of the hyoid the UES^l^ opening (*P* = 0.066) No significant changes in anterior movement and the PAS Group 2. No significant increase in all biomechanical outcome measures No significant changes in the PAS

Lim et al. (2009) [20]	Randomized controlled study	Group (ES + TTS): *n* = 16 Group 2 (TTS): *n* = 12	Group 1 Subcortical = 12 Brainstem = 2 Hemispheric = 2 Group 2 Subcortical = 8 Brainstem = 2 Hemispheric = 2	Group 1: 20 sessions Group 2: 20 sessions	Intensity: approximately 7 mA Duration: 60 minutes a day for 5 days a week Sensory level	Digastric, mylohyoid, and thyrohyoid muscles	Group 1. Difference in PAS^j^ scores after treatment (semisolid and liquid) = 2 and 2.5 (*P* < 0.05) Difference in swallow function scores after treatment = 2 (*P* < 0.05) Difference in PTT^k^ after treatment (semisolid and liquid) = 0.11 and 0.10 (*P* < 0.05) Group 2. Difference in PAS scores after treatment (semisolid and liquid) = 0 and 0 (*P* > 0.05) Difference in swallow function scores after treatment = 0 (*P* > 0.05) Difference in PTT after treatment (semisolid and liquid) = 0.01 and 0.02 (*P* > 0.05)

Gallas et al. (2010) [29]	Outcome study	One group (ES): *n* = 11 patients with chronic poststroke dysphagia	Hemispheric = 7 Brainstem = 4	5 sessions	Frequency: 80 Hz Mean intensity: 4.8 V (sensory threshold) Duration: 60 minutes a day for 5 consecutive days Sensory level	Submental muscles	Initial average of dysphagia handicap index scores = 31 ± 7 Posttreatment average of dysphagia handicap index scores = 23 ± 7 (*P* < 0.05) Aspiration and residue scores decreased after ES (*P* < 0.05) The swallow response time decreased after ES (*P* < 0.05)
